# Therapeutic Role of Functional Components in Alliums for Preventive Chronic Disease in Human Being

**DOI:** 10.1155/2017/9402849

**Published:** 2017-02-05

**Authors:** Yawen Zeng, Yuping Li, Jiazhen Yang, Xiaoying Pu, Juan Du, Xiaomeng Yang, Tao Yang, Shuming Yang

**Affiliations:** ^1^Biotechnology and Genetic Resources Institute, Yunnan Academy of Agricultural Sciences/Agricultural Biotechnology Key Laboratory of Yunnan Province, Kunming 650205, China; ^2^Yuxi Agriculture Vocation-Technical College, Yunnan, Yuxi 653106, China; ^3^Kunming Tiankang Science & Technology Limited Company, Yunnan, Kunming 650231, China

## Abstract

*Objectives*. Functional components in alliums have long been maintained to play a key role in modifying the major risk factors for chronic disease. To obtain a better understanding of alliums for chronic disease prevention, we conducted a systematic review for risk factors and prevention strategies for chronic disease of functional components in alliums, based on a comprehensive English literature search that was conducted using various electronic search databases, especially the PubMed, ISI Web of Science, and CNKI for the period 2007–2016.* Allium* genus especially garlic, onion, and Chinese chive is rich in organosulfur compounds, quercetin, flavonoids, saponins, and others, which have anticancer, preventive cardiovascular and heart diseases, anti-inflammation, antiobesity, antidiabetes, antioxidants, antimicrobial activity, neuroprotective and immunological effects, and so on. These results support* Allium* genus; garlic and onion especially may be the promising dietotherapeutic vegetables and organopolysulfides as well as quercetin mechanism in the treatment of chronic diseases. This review may be used as scientific basis for the development of functional food, nutraceuticals, and alternative drugs to improve the chronic diseases.

## 1. Introduction

Natural products, which associate with health foods for plant origin centers, are very popular to prevent various chronic diseases. Asia is one of the origin centers of* Allium* genus, especially garlic (*Allium sativum* L.), onion (*Allium cepa* L.), and Chinese chive (*Allium tuberosum*) [1]; however sulfur compounds from alliums have played a key roles in defense [2]. Sulfur is the component of some amino acids and Fe-S clusters for enzymes activity [3]. Fe-S clusters are very important for origin of life, especially acetyl-CoA, DNA, and RNA world [4]. Chronic diseases are a serious threat to human health and economic growth; however the costs of five chronic diseases (cardiovascular diseases, cancers, diabetes, chronic respiratory disease, and mental illness) will be over US$47 trillion from 2011 to 2030 [5, 6]. Garlic, which was one of the best foods for preventing chronic disease, has been utilized in treating human diseases since 5000 years [7–9]. The high consumption of* Allium* genus reduced the risk for various cancers [10, 11].* Allium* oils had the highest antibacterial activity or the strongest antioxidants [12]. The consumption of onion extract had better cardioprotective effect by antioxidation and anti-inflammation, attenuating cholesterol and cardiovascular disease [13].

It is known that* Allium* genus are widely cultivated and used all over the world, particularly garlic, onion, shallot (*Allium ascalonicum*), leek (*Allium ampeloprasum*), and chive (*Allium schoenoprasum*) [14]. The edible species of* Allium* L. in China included 68 species and 6 varieties, from which there are 7 cultivated species and 2 varieties and 61 wild species and 4 varieties [15]. China is not only the largest production and export but also the consumer country for garlic and onion in the world, such as the annual output and export of garlic accounted for more than 70% of the world. Chinese chive is widely cultivated in Asia especially China, from which 2,125 SSRs are identified, which will facilitate its genetic research [16].

Our review is an overview on the scientific literature concerning the effects of functional components in* Allium* genus especially garlic and onion in the prevention or treatment of chronic disease in Human Being.

## 2. Functional Components in* Alliums*

Functional components of food can be applied in the treatment and prevention of chronic diseases; they include nonstarchy carbohydrates (dietary fibres, resistant starch, and fucoidan), antioxidants (organosulfur compounds, polyphenols, carotenoids, tocopherols, tocotrienols, phytosterols, and isoflavones), unsaturated fatty acids, bioactive peptides, sterols, and phytoestrogens [17].* Allium* genus is rich in sulfur compounds, steroidal saponins, flavonoids, and so on, which have anticancer, antioxidant, antiplatelet aggregation, antiatherosclerosis, antimicrobial, and lower blood lipids and blood glucose biological activity.* S*-alk(en)yl-l-cysteine sulfoxides are cysteine-derived secondary metabolites highly accumulated in the genus* Allium* [18]. Structures of 15 major color compounds of onion and leek were determined; the pigment is a complex mixture of highly conjugated species composed of two N-substituted 3,4-dimethylpyrrole-derived rings linked by bridge from the methyl group of methiin or propenyl group of isoalliin [19].

### 2.1. Garlic

Garlic is a very important source of dietary for antioxidant properties, including sulfur compounds, polyphenols, and carotenoids. Garlic contains sulfur compounds like diallyl thiosulfinate (allicin), diallyl trisulfide, allyl methyl trisulfide, diallyl disulphide, ajoene, and others, which show anticancer, antioxidation, anti-inflammation, immunomodulatory, antimicrobial, hypoglycemic, and cardiovascular protections [20]. Allicin from garlic with many biological activities is a consequence of the molecule's chemistry [21]. Garlic has four major organosulfur compounds:* S*-allylcysteine, alliin,* S*-methylcysteine, and* S*-ethylcysteine [7].* S*-allylcysteine has antioxidation, regulated redox, antiapoptotic, anti-inflammation, proenergetic, and signaling capacities [22]; however it ameliorates lots of diseases [23].* S*-allylmercaptocysteine with anticancer activity inhibits the growth of cancer cells [24]. Diallyl disulfide can prevent tumorigenesis by inhibiting inflammation and GSK-3*β* [25] as well as NF-*κ*B mediated pathway in human leukemia [26].* S*-propargyl-cysteine, a structure of analog* S*-allylcysteine, will be discovered in chronic diseases because it activated antioxidation, inhibited STAT3, elevated p53/Bax, and decreased Ca^2+^ accumulation and inflammatory cytokines [27]. Garlic reduces the risks of cardiovascular disease by inhibiting platelet aggregation and lowering cholesterol and blood pressure, based on the fact that allicin (diallyl thiosulfinate) is degraded into diallyl polysulfides by H_2_S preventing myocardial injury and dysfunction [9].* AsFMO1* in garlic for* S*-allyl-L-cysteine* S*-oxygenase contributes to the production of alliin through the conversion and biosynthesis of *γ*-glutamyl-*S*-allyl-L-cysteine in storage leaves [18]. The antiwrinkle for skin aging of caffeic acid,* S*-allyl cysteine, and uracil associated with antioxidation and anti-inflammation by modulating MMP via NF-*κ*B signaling [28]. The* S*-ethyl cysteine or* S*-methyl cysteine can protect bronchial cells and respiratory epithelia [29]. Chemical constituents of garlic were alliin (0.320%), allicin (0.273%), and allitride (0.357%) [30]. The black garlic allicin had the function of reducing blood glucose, and the main function of substance contained 7 sulfide materials, in which the total content was 2.279% [31]. In garlic, cycloalliin level at 80°C showed the highest yield (5.05 mmol/mL), among the tested temperatures [32]. Sucrose methyl 3-formyl-4-methylpentanoate from garlic has antimicrobial activities by enhanced microbial killing and blockage of cytokine storm [33]. The glycoprotein from ripe garlic has potential uses in functional foods and medical applications by the 1,1-diphenyl-2-picrylhydrazil free radical scavenging activity and polyunsaturated fatty acid inhibiting ability [34]. *β*-Carotene (73.44 *μ*g/g) accumulation in the leaves of garlic with synthase and desaturase of phytoene is a significantly higher than that of other organs [35]. The garlic bulbs extract had ten furostanol saponins, ten types of voghierosides, two eugenol diglycosides, agigenin 3-O-trisaccharide, and gitogenin 3-O-tetrasaccharide [36].

### 2.2. Onion

Onion (*Allium cepa*) is an important source of dietary phytochemicals with proven antioxidant properties, such as organosulfur compounds, phenolic acids, flavonoids, thiosulfinates, and anthocyanins. 30 compounds from volatiles compounds of black onion were identified, which accounted for 52.63% of all compounds and 81.69% of the total peak areas; these components included 19 sulfur-containing volatiles compounds, such as diallyl sulfide, methyl allyl sulfide, 3-hydroxysulfolane, 2,4-dimethylthiophene, 2-methoxythiophene, 1,4-dithiane, and 1,3-dithiane [37]. A total of 49 types of volatile compounds were identified from onion, which were mainly sulfur compounds, alcohols, aldehyde, ester, and other chemical groups, but there was significant difference in volatile compound pattern and their relative contents from fresh and dried onion [38]. The diabetic rats supplemented with either onion or with single components (alliin, allitride, and* S*-methylcysteine sulfoxide) possess lowering plasma glucose concentrations and body weight [39] and preventive cardiovascular diseases. The major components of onion were quercetin, quercetin glucosides, isorhamnetin glucosides, and kaempferol glucoside; their concentrations in Tropea were 20–230-fold higher than that of Montoro, but total anthocyanins in Montoro were 30-fold higher than that of Tropea [40]; however quercetin and catechins were with antiatherosclerotic effect [41]. Onion peel extract is an important ingredient for nutraceuticals and functional foods based on p-coumaric acid, vanillic acid, epicatechin, and morin [42].

### 2.3. Chinese Chive

Chinese chive (*Allium tuberosum*) is an important source of dietary phytochemicals with proven antioxidant properties, such as organosulfur compounds, flavonoids, and saponins. 47 Compounds of the essential oil in wild Chinese chive were identified, which included 28 sulfur-containing compounds, 4 aldehydes, 3 alcohols, 3 ketones, and hydrocarbons [43].* Allium tuberosum* Rottler has two new phenylpropane glycosides and four known flavonoids (kaempferol 3-O-*β*-sophoroside; 3-O-*β*-D-(2-O-feruloyl)-glucosyl-7,4-di-O-*β*-D-glucosyl-kaempferol; 3-O-*β*-sophorosyl-7-O-*β*-D-(2-O-feruloyl) glucosyl-kaempferol; and kaempferol 3,4′-di-O-*β*-D-glucoside) [44]. The major components and percentage of volatile oil were 39.31% diallyl disulphide, 32.76% disulfide-methyl-2-propenyl, and 12.16% trisulfide-di-2-propenyl; Chinese chive volatile oil showed extensively potential ability for neotype antibacterials and food preservative [45]. The volatiles of Chinese chive contained allyl methyl sulfide and diallyl disulfide [46].* Allium tuberosum* is an important functional foods with antitumor, anti-inflammation, nerve protection, and antioxidant activity; nine compounds which were isolated from the roots were identified as 4,8-dihydroxyacetophenone-8-O-ferulate; 4,8-dihydroxyacetophenone; 3,4,5-trimethoxybenzoic acid; 3,4,5-trimethoxycinnamic acid; buddlenol D;* E*-1,6,11-triene-4,5,9-trithiadodeca-9,9-dioxide; tianshic acid; daucosterol; and linoleic acid [47]. A new phenylpropanoid glucoside tuberosinine D and a chain compound (Z)-11R,12S,13S-trihydroxy-9-octadecenoate were isolated from the roots of* Allium tuberosum* [48]. Six spirostanol saponins from the roots of* Allium tuberosum*, among them are 25(S)-Schidigera-saponin D5, shatavarin IV, and new saponin 2, showed antibacterial activities against* Bacillus subtilis* and* Escherichia coli* [49].

## 3. Chronic Disease Prevention of* Allium* Genus

In Table 1 are summarized the studies reporting the functional components for preventing chronic diseases in* Alliums* genus.

### 3.1. Functional Components and Anticancer of the* Allium* Genus

Functional components in garlic of anticancer effect included allicin [diallyl thiosulfinate],* S*-allylmercaptocysteine,* S*-propargyl-L-cysteine,* S*-benzyl-cysteine, polysulfanes, diallylpolysulfides, allyl mercaptan, and Z-ajoene; nevertheless, major functional components in onion of anticancer effect contained onionin A, fisetin, diosgenin, and quercetin. Moreover, major functional components in Chinese chive of anticancer effect were thiosulfinates and tuberoside M (see Table 1). These data showed that functional components and molecular mechanism for anticancer effect had significant difference among garlic and onion as well as Chinese chive, but it is necessary to conduct future research on similar functional components and molecular mechanism for anticancer effect in* Allium* genus.

#### 3.1.1. Garlic

Allicin from garlic can inhibit the development and metastasis of colorectal cancer, based on improving the immune function and inhibiting tumor vessel formation as well as the expression of surviving gene, so as to promote the apoptosis of cancer cells [50]. Allicin inhibits H_2_O_2_-induced senescence in human umbilical vein endothelial cells through activation of* SIRT1* [51]. Allicin can improve pancreatic cancer therapy which would reverse gene silencing and suppress cancer cell growth [52]. Allicin from garlic can inhibit the proliferation and induce the apoptosis of MGC 803 human gastric carcinoma cells, which may be achieved through the enhanced expression of p38 and cleaved caspase 3 [53]. JNK activation and mitochondrial Bax translocation are involved in allicin-induced apoptosis in human ovarian cancer SKOV3 cells [54]; however, allicin and* S*-allylmercaptocysteine as well as* S*-propargyl-L-cysteine were one of the novel remedies which possess lots of human antitumor activities including neuroblastoma, adenocarcinoma, and breast cancer [55–57]. Garlic has been used as treatment for benign prostatic hyperplasia [109].* S*-benzyl-cysteine in aged garlic extract is a structural analog of* S*-allylcysteine, which activated the mitochondrial-dependent apoptosis through p53 and Bax/Bcl-2 pathways in human gastric cancer cells [58]. The diallyl trisulfide, diallyl tetrasulfide, diallyl sulfide, and diallyl disulfide from garlic have various anticancer activities such as affecting the gene expression of human colon cancer cells [59, 60]. The allyl mercaptan with the best histone deacetylase inhibitor from garlic revealed its anticancer mechanisms [61]; however Z-ajoene from garlic can be treated the glioblastoma by targeting its cancer stem cells [62]. Garlic intake is negatively associated with the cancer and cardiovascular disease, based on activation of genes with immunity, apoptosis, and xenobiotic metabolism [110].

#### 3.1.2. Onion

Onionin A from onions is considered useful for the additional treatment of patients with ovarian cancer owing to its suppression of the protumor activation of tumor-associated macrophages and direct cytotoxicity against cancer cells [63]. The flavonoid fisetin (3,7,3,4-tetrahydroxyflavone) from onion is a promising agent for cancer treatment [64]. Onion can be used to improve hyperglycemia and insulin resistance in breast cancer in chemotherapy [111]. The quercetin and diosgenin of onion can be used as a good anticancer therapy by small ligand molecules for targeting neuropilin-1 receptor and inhibiting growth of various cancer cells [65, 66].

#### 3.1.3. Chinese Chive

The thiosulfinates from Chinese chive inhibited the proliferation and activation of human colon cell by the caspase-independent apoptotic pathways [67]. Tuberoside M from seeds of Chinese chive has a significant effect for inhibiting the growth of human leukemia cells [68].

### 3.2. Functional Components and Preventing Cardiovascular Diseases of the* Alliums* Genus

Functional components in garlic of preventing cardiovascular diseases included allicin, allyl sulfides, flavonoids, and polysulfides; nevertheless, major functional components in onion of preventing cardiovascular diseases contained quercetin, flavonoids, furostanol saponins, and thiosulfinates. Moreover, major functional components in Chinese chive of preventing cardiovascular diseases were quercetin, flavonoids, furostanol saponins, and thiosulfinates (see Table 1). These data showed that functional components and molecular mechanism for preventing cardiovascular diseases effect had significant difference between garlic and onion, but it is necessary to conduct future research on similar flavonoids and their molecular mechanism for preventing cardiovascular diseases in* Allium* genus.

#### 3.2.1. Garlic

Lots of organosulfur compounds (allicin, allyl sulfides) from garlic are responsible for the food function of prevention of cardiovascular diseases (cancer, hypertension, dyslipidemia, obesity, and hyperglycemia) [69]. Garlic can play an important role in preventing atherosclerosis and cardiovascular protection based on risk factor reduction of hypertension and total cholesterol as well as surrogate markers [112, 113]. The garlic bulbs of “74-x” had the highest phenolic content (total phenolic, flavonoids), which associated with the strongest antioxidant and protection abilities [70]. H_2_S is a key signaling molecule that induces the relaxation and vasodilation of smooth muscle cell and lowering blood pressure [71]. Daily garlic intake is a predictor of endothelial function for patients with ischemic stroke and preventing atherosclerotic [114].

#### 3.2.2. Onion

The flavonoids and quercetin in onion could be recommended for preventing and treating various cardiovascular diseases by controlling cholesterol level and enhancing antioxidation ability [72, 73]. The seeds of onion with eight new furostanol saponins can treat diarrhea and promote blood flow of human population for Uygur nationality in China [74]. The thiosulfinates of onion with antiplatelet agents can prevent cardiovascular diseases [75].

#### 3.2.3. Chinese Chive

Chinese chive leaf water-soluble substances (amino-styrene-acrylic acids and their glycosides) have a strong anticoagulant effect, while the butanol extract promotes the coagulation of the blood, in favor of understanding the reasons of traditional Chinese medicine about* Allium tuberosum* with invigorating the circulation of blood and hemostasis [76].

### 3.3. Functional Components and Preventing Heart Diseases of the* Allium* Genus

Functional components in garlic of preventing heart diseases included allicin,* S*-propargyl-cysteine, garlic oil, and polysulfides; nevertheless, major functional components in onion of preventing heart diseases contained quercetin (see Table 1). These data showed that functional components and molecular mechanism for preventing heart diseases had significant difference among garlic and onion, but it is necessary to conduct future research on similar components and their molecular mechanism for preventing cardiovascular diseases in* Allium* genus.

#### 3.3.1. Garlic

Allicin from garlic may exhibit antimyocardial fibrosis effect and the mechanism related to TGF *β*/Smads signal transduction [77].* S*-propargyl-cysteine has shown cardioprotection in ischemic heart disease [27]. Garlic-derived polysulfides may be useful in the treatment of myocardial ischemic disease [78]. Garlic exhibits cardioprotective properties against cardiotoxicity, arrhythmia, hypertrophy, ischemia-reperfusion injury, cardiac and mitochondrial dysfunction, and myocardial infarction [115, 116].

#### 3.3.2. Onion

The quercetin with cardioprotection from onion skin reduces ambulatory blood pressure in hypertension patients [79]. Onion extract (10 g/kg) decreased the infarct size and cell death of heart [117].

### 3.4. Functional Components and Anti-Inflammation of the* Alliums* Genus

Functional components in garlic of anti-inflammation included allicin,* S*-propargyl-cysteine, and diallyl trisulfide; nevertheless, major functional components in onion of anti-inflammation contained quercetin-3-O-glucoside (see Table 1). These data showed that functional components and molecular mechanism for anti-inflammation had significant difference between garlic and onion, but it is necessary to conduct future research on similar components and their molecular mechanism for anti-inflammation in* Allium* genus.

#### 3.4.1. Garlic

The garlic and onion showed the anti-inflammatory and antioxidant effects [118]. Allicin from garlic displays a significant protective effect against EA.hy926 endothelial cell injury induced by PM2.5 and its mechanism may be related to the attenuations of inflammation and oxidative stress via the inhibition of ERK1/2 pathway [80]. Allicin from garlic may be useful in reducing oxidative stress, inflammation, vascular dysfunction, and the aortic pathology [81, 82].* S*-propargyl-cysteine has shown anti-inflammation in acute pancreatitis [27]. Diallyl trisulfide in five organosulfur compounds from garlic is not only a highly promising therapeutic candidate for treating inflammation-related neurodegenerative diseases [83], but also has varied potential therapeutic activities, especially periodontal inflammation [84], which revealed anti-inflammatory effect by downregulating AKT1/TGF-*β*-activated kinase-mediated NF*κ*B and MAPK signaling pathways [85].

#### 3.4.2. Onion

Onion stalk extract can be used as reducing atherosclerosis and regulating inflammatory response [119]. Eicosapentaenoic acid combined quercetin-3-O-glucoside from onion had significant anti-inflammation and hypolipidemic effects, which can be used as the treatment of obesity-related diseases [86].

### 3.5. Functional Components and Antiobesity of the* Allium* Genus

Functional components in garlic of antiobesity included diallyl disulfide and* S*-methyl L-cysteine; nevertheless, major functional components in onion of antiobesity contained* S*-methyl L-cysteine and quercetin (see Table 1). These data showed that functional components and molecular mechanism for antiobesity had similar organosulfur compounds between garlic and onion.

#### 3.5.1. Garlic

Garlic can reduce body weight and fat mass among subjects [120]; meanwhile 20 mg/kg diallyl disulfide was effective in preventing nonalcoholic fatty liver disease [87]. Garlic compared with placebo can not only effectively reduce serum lipids and body mass index in elderly hyperlipidemic patients [121], but also minimised the high-fat-diet-induced fatty liver changes in rats [122]. Administration of garlic plus lemon juice resulted in an improvement in lipid levels, fibrinogen, and blood pressure of patients with hyperlipidemia [123].* S*-methyl L-cysteine from garlic and onion found to be effective in improving the high fructose induced hyperglycemia and dyslipidemia [88].

#### 3.5.2. Onion

Onion consumption combined with healthy diet can be effective in nonalcoholic fatty liver disease management [124]. Quercetin in onion peel could not only ameliorate the flow-mediated dilation and circulating endothelial progenitor cells, antiobesity by suppressing preadipocyte differentiation and inhibiting adipogenesis, and genes regulating intracellular lipolysis, but also has anticholesterol, antithrombotic, and insulin-sensitizing properties [89–91].

### 3.6. Functional Components and Antidiabetes of the* Alliums* Genus

Functional components in garlic of antidiabetes included* S*-methyl L-cysteine,* S*-allyl cysteine, diallyl disulphide, and* S*-allyl-mercapto-captopril; nevertheless, major functional components in onion of antidiabetes contained S-methylcysteine and flavonoids (see Table 1). These data showed that functional components and molecular mechanism for antidiabetes had similar organosulfur compounds between garlic and onion.

#### 3.6.1. Garlic

*S*-methyl L-cysteine from garlic and onion could treat and manage the diabetes [88]; however* S*-allylcysteine might be stimulating the synthesis of insulin through circulating thyroid hormones [92]. Diallyl disulphide of garlic oil has the hypolipidemic effect and reducing the morbidity in diabetes [93]. The* S*-allyl-mercapto-captopril for a promising antidiabetic and cardiovascular protective agent integrates the antihypertensive feature between allicin and captopril [94]. Garlic plays an important role of improving metabolic syndrome containing abdominal obesity, hypertension, dyslipidemia, and hyperglycemia disorders [125], which prevents obesity by the downregulation of gene expression patterns [126].

#### 3.6.2. Onion

*S*-methylcysteine and flavonoids of onion can decrease the levels of blood glucose, serum lipids, oxidative stress, and lipid peroxidation, meanwhile increasing insulin secretion and antioxidant enzyme activity [95]. The ethanolic extract of onion can control diabetes by the phosphatidylinositol-4,5-bisphosphate 3-kinase/Akt dependent pathway [127].

### 3.7. Functional Components and Neuroprotective Effects of the* Allium* Genus

Functional components in garlic of neuroprotective effects included allicin,* S*-methylcysteine,* S*-allyl cysteine,* S*-allyl-L-cysteine, diallyl disulfide, diallyl trisulfide, N-*α*-(1-deoxy-D-fructose-1-yl)-L-arginine, and Z-ajoene; nevertheless, major functional components in onion of neuroprotective effects contained flavonoids and quercetin (see Table 1). These data showed that functional components and molecular mechanism for neuroprotective effects had significant difference among garlic and onion, but it is necessary to conduct future research on similar components and their molecular mechanism for neuroprotective effects in* Allium* genus.

#### 3.7.1. Garlic

Allicin from garlic can be used as a neuroprotective strategy for ischemic stroke [96]; however* S*-propargyl-cysteine and* S*-methyl cysteine were the effective neuroprotective agents [27, 128].* S*-allyl-L-cysteine from garlic against neuronal cell death of endoplasmic reticulum stress is inhibiting calpain by interaction with its Ca^2+^-binding site [129]; meanwhile diallyl disulfide possess the hippocampal neurogenesis and neurocognitive functions through modulating ERK and BDNF-CREB signaling [97]. Diallyl trisulfide in garlic oil has lots of neuroprotective effects in transgenic mice [98]. Aged garlic extract and its N-*α*-(1-deoxy-D-fructos-1-yl)-L-arginine could attenuate neuroinflammatory and promote resilience in lipopolysaccharide-activated cells [130]. Z-ajoene from garlic oil and aged garlic extract could promote neuroprotective effects by reducing lipid peroxidation and antioxidant or anti-inflammatory activities [99, 131].

#### 3.7.2. Onion

Flavonoids extracted from onion could ameliorate symptoms of intracerebral hemorrhage by inhibiting activation of microglia and relieve proinflammatory factors of hematoma [100]; however quercetin could protect brain cells against oxidative stress for Alzheimer's disease and neurodegenerative disorders [101]. The onion extract could prevent the blood-brain barrier during brain ischemia [132].

### 3.8. Functional Components and Immunological Effects of the* Alliums* Genus

Functional components of immunological effects included allyl methyl disulfide and so on. Allyl methyl disulfide from fresh garlic can treat the immunological disorders, such as ulcerative colitis, Crohn's disease, and intestinal inflammation [53]. Lipid garlic extract as an immunotherapy has better cure rates for the recalcitrant multiple common warts [133]. Fresh aqueous garlic and onion extracts play an important role in enhancing immune function [102].

### 3.9. Functional Components and Antimicrobial Activities of the* Allium* Genus

Functional components in garlic of antimicrobial activities included allicin, thiosulfinates, diallyl monosulfide, diallyl disulfide, diallyl trisulfide, diallyl tetrasulfide, and ajoene; nevertheless, major functional components in onion of antimicrobial activities contained saponins and Ace-AMP1 (see Table 1). These data showed that functional components and molecular mechanism for antimicrobial activities had significant difference among garlic and onion, but it is necessary to conduct future research on similar components and their molecular mechanism for neuroprotective effects in* Allium* genus.

The components of antimicrobial activities (bacteria, fungi, viruses, and parasites) of garlic are allicin, thiosulfinates, and others [103, 104]. Antimicrobial activity of allicin was a thiol reagent [105]; however garlic oils showed a good antimicrobial activity against* Staphylococcus aureus*,* Pseudomonas aeruginosa*, and* Escherichia coli*, based on diallyl monosulfide, diallyl disulfide, diallyl trisulfide, and diallyl tetrasulfide [106]. Allicin and ajoene as well as oil from garlic showed significant antimycobacterial and antibacterial activity [20]. Garlic oil has the anti-influenza virus activities in mice [134].

The chemical structure of five new saponins compounds (persicosides A and B, persicosides C1/C2 and D1/D2, and persicoside E) from Persian leek as well as three saponins (ceposides A, B, and C) from the bulbs of white onion were identified, while persicosides (A, B) and ceposides (A, B, and C) showed the higher antifungal activity [36, 107]. Ace-AMP1 is a potent antifungal peptide found in onion seeds and could be used as an effective fungicide [108].

### 3.10. Functional Components and Other Effects of the* Alliums* Genus

*γ*-Glutamyl-*S*-allyl-cysteine peptide of fresh garlic involves the radical scavenging and metal-chelating capacities [135]. Garlic + onion can remarkably decrease in liver steatosis, serum liver enzymes, oxidative markers, and lipid peroxidation than that of vegetables alone [136]. Garlic saponins can protect dPC12 cells from hypoxia damage [137].

## 4. Major Mechanisms and Structural Activity of Alliums Compounds for Preventive Chronic Disease

### 4.1. Organopolysulfides Mechanism and Its Structural Activity

**Figure 1:**
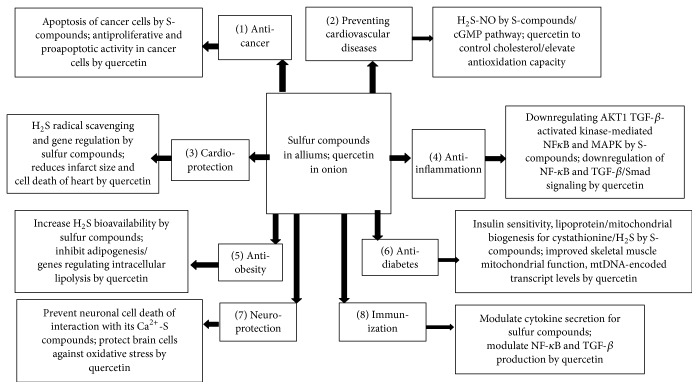
Possible mechanisms of functional components in allium for preventive chronic disease.

Major mechanisms of alliums organopolysulfides for preventive chronic disease include anticancer, preventive cardiovascular and heart diseases, anti-inflammation, antiobesity, antidiabetes, antimicrobial activities, and neuroprotective and immunological effects (Figure 1). Hydrogen sulfide (H_2_S) is a gaseous signaling molecule; however, the polysulfides as H_2_S donors have established the relationship between structure and health promotion activity [138]. Preventive mechanism for some chronic diseases of garlic and onion is an active mechanism of many therapeutic effects such as cardiovascular disorders, obesity, metabolic syndrome, gastric ulcer, and even cancer by modulating cytokine secretion with immunomodulation and anti-inflammatory effects for sulfur-containing compounds [139]. Anticancer mechanism of garlic and onion is the induction of apoptosis for cancer cells by sulfur-containing compounds [140]. The mechanisms of polysulfides in cardioprotection are as follows: H_2_S releasing, radical scavenging, and gene regulation with enzyme pathways [138]. ZYZ-803 with therapeutic cardiovascular diseases is a H_2_S-NO conjugated donor developed by* S*-propyl-L-cysteine and furoxan; based on this it can regulate vascular tone through cGMP pathway [141]; H_2_S is biosynthesized by three enzymes from L-cysteine and homocysteine; NO is generated endogenously from L-arginine by the action of various isoforms of NOS; two gases have been transpired as the key and independent regulators of cardiovascular, nervous, gastrointestinal, respiratory, and immune systems [142]. Obesity in mice reduced the H_2_S bioavailability; the depletion of macrophage H_2_S increases the store-operated Ca^2+^ entry through disinhibition of Orai 3 and promotes the production of proinflammatory cytokines [143]. Cystathionine *γ*-lyase-derived H_2_S affects lipoprotein synthesis, insulin sensitivity, and mitochondrial biogenesis, in which tissue-specific regulation pathway might be a promising therapeutic target of diabetes and other metabolic syndromes [144]. The ameliorative action of organopolysulfides in garlic on the elevated blood pressure and renal clearance functions in diabetes may be mediated through attenuating modulations in plasma and kidney angiotensin I converting enzyme type-1 and angiotensin II concentrations; thiosulfinate in garlic is a promising agent for the management of postprandial hyperglycemia [145, 146].

### 4.2. Quercetin Mechanism and Its Structural Activity

Major mechanisms of alliums compounds especially quercetin for preventive chronic disease include anticancer, preventive cardiovascular and heart diseases, anti-inflammation, antiobesity, antidiabetes, antimicrobial activities, and neuroprotective and immunological effects (Figure 1). Quercetin in onion is not only a bioflavonoid with antiproliferative and proapoptotic activity in many cancer cells [147] but also has neuroprotective effects by stimulating cellular defenses against oxidative stress [148] and has combating various cardiovascular diseases by suppressing cholesterol level and elevate total antioxidation capacity [72]. Quercetin in onions increases insulin sensitivity and adiposity and improves glucose tolerance, based on improved skeletal muscle mitochondrial number and function, and mtDNA-encoded transcript levels [149]. Daily quercetin supplementation is associated with antioxidative and anti-inflammatory potential and downregulation of NF-*κ*B and TGF-*β*/Smad signaling, probably via interference with TLR signaling [150]. Onions extract and quercetin with downregulated NF-*κ*B pathway can inhibit RANKL/*Porphyromonas gingivalis* LPS-induced osteoclastogenesis under inflammatory conditions via attenuation of NF-*κ*B activation [151]. Orally administered quercetin has antihypertensive effect and is responsible for vasorelaxant activity [152]. The protective mechanisms of onions extract and its quercetin against BSO-induced oxidative stress in neuronal cells associate with the inactivation of PKC-*ε* induced by phosphorylating ERK1/2 [153]. Quercetin has an effective therapeutic strategy for patients with liver damage and fibrosis of immune response, based on association with its ability to modulate NF-*κ*B and TGF-*β* production [154].

## 5. Conclusion Remarks and Future Perspectives

The data summarized in the current review point out that major mechanism and many functional components derived from* Allium genus*, especially garlic and onion, exert potent preventing chronic diseases. Although functional components in* Alliums* for preventing and treating chronic diseases seem a complicated task, the development of functional foods may still open new venues for therapeutic interventions. Regular consumption of such functional foods for* Allium* may become a successful and safe strategy to treat chronic disease conditions. These data support that* Allium* genus especially garlic and onion is rich in organosulfur compounds and quercetin, which are known to play a pivotal role in many chronic diseases.

This review provides useful information that will guide future research, which will provide strategies for efficient, organosulfur-based prevention, or treatment, of chronic diseases. Further efforts are needed to resolve several remaining hurdles, such as a better understanding of the interconnection between functional components and preventing chronic diseases in clinical trials. Further studies are necessary to unravel the key compounds for preventing and treating chronic disease of other* Allium* genus except garlic and onion, as these compounds present various mechanistic actions and their clinical applications need to be tested. This review may be used as a starting point for novel nutraceuticals, functional foods, or complementary and alternative drugs to maintain or improve the chronic diseases.

## Figures and Tables

**Table 1:** Functional components for preventing chronic disease in garlic, onion, and Chinese chive.

Chronic disease	Alliums	Functional components	Ref.
Anticancer	Garlic	Allicin[diallyl thiosulfinate]; *S*-allylmercaptocysteine; *S*-propargyl-L-cysteine; *S*-benzyl-cysteine; polysulfanes; diallylpolysulfides; allyl mercaptan; Z-ajoene;	[50–62]
	Onion	onionin A; fisetin; diosgenin; quercetin	[63–66]
	Chinese chive	thiosulfinates; tuberoside M	[67, 68]

Preventive cardiovascular diseases	Garlic	Allicin, allyl sulfides; flavonoids; polysulfides;	[69–71]
	Onion	quercetin; flavonoids; furostanol saponins; thiosulfinates	[72–75]
	Chinese chive	Glycosides; amino-styrene-acrylic acids	[76]

Preventive heart diseases	Garlic	*S*-Propargyl-cysteine; allicin; garlic oil; polysulfides	[27, 77, 78]
	Onion	Quercetin	[79]

Anti-inflammation	Garlic	*S*-Propargyl-cysteine; allicin; diallyl trisulfide	[27, 80–85]
	Onion	Quercetin-3-O-glucoside	[86]

Antiobesity	Garlic	Diallyl disulfide; *S*-methyl L-cysteine	[87, 88]
	Onion	*S*-Methyl L-cysteine; quercetin	[88–91]

Antidiabetes	Garlic	*S*-Methyl L-cysteine; *S*-allylcysteine; diallyl disulphide; *S*-Allyl-mercapto-captopril	[88, 92–94]
	Onion	*S*-Methylcysteine; flavonoids	[95]

Neuroprotective effects	Garlic	N-*α*-(1-Deoxy-D-fructos-1-yl)-L-arginine; *S*-methylcysteine; allicin; *S*-allylcysteine; *S*-allyl-L-cysteine; diallyl disulfide; diallyl trisulfide; Z-ajoene	[22, 27, 96–99]
	Onion	Flavonoids; quercetin	[100, 101]

Immunological effects	Garlic	Allyl methyl disulfide	[53]
	Onion	Onion extracts	[102]

Antimicrobial activities	Garlic	Allicin; thiosulfinates; diallyl monosulfide; diallyl disulfide; diallyl trisulfide; diallyl tetrasulfide; ajoene	[20, 103–106]
	Onion	Saponins; Ace-AMP1	[36, 107, 108]
